# GWAS-follow-up Studies Identified a Connection between Abnormal LIF/JAK2/STAT1 Signaling and Overproduction of Galactose-Deficient IgA1 in the Tonsillar IgA1-Secreting Cells from Patients with IgA Nephropathy

**Published:** 2024-01-22

**Authors:** Koshi Yamada, Jan Novak, Yusuke Suzuki

**Affiliations:** 1 Department of Nephrology, Juntendo University Faculty of Medicine, Tokyo, Japan; 2 Department of Microbiology, University of Alabama at Birmingham, Birmingham, Alabama, USA

## DESCRIPTION

IgA Nephropathy (IgAN) is a common primary glomerulonephritis with its characteristic IgA1-containing glomerular immunodeposits. Patients with IgAN have a high rate of progression to kidney failure; up to 40% of patients reach that stage within 20–30 years since the diagnosis, despite receiving optimized standard care.

A suspected connection between IgAN and the mucosal immune system is highlighted by the fact that the main production sites of IgA1 reside in the mucosal tissues, and that a common clinical feature at the onset of IgAN is macroscopic hematuria with a concurrent upper respiratory-tract infection, i.e., synpharyngitic hematuria [1,2].

The “multi-hit hypothesis” has been proposed to explain the pathobiology of IgAN [3], wherein Galactose-deficient IgA1 (Gd-IgA1) glycoforms are recognized by Gd-IgA1-specific IgG autoantibodies to form immune complexes. These complexes bind other proteins, such as complement C3, and some of the resultant complexes may deposit in the glomeruli and induce kidney injury. Notably, serum levels of Gd-IgA1 and IgG autoantibodies are predictive of disease progression [4–7]. Although the precise location of the cells producing Gd-IgA1 *in vivo* is still under investigation, tonsillar B cells have been proposed to be significant producers of Gd-IgA1, potentially explaining why tonsillectomy improves clinical symptoms of some IgAN patients [8,9].

We previously demonstrated that Interleukin-6 (IL-6), a pro-inflammatory cytokine with multiple roles in immune responses, selectively increases the production of Gd-IgA1 in IgA1-producing cell lines from IgAN patients; this process is mediated by an abnormal activation of STAT3 [10]. Although serum Gd-IgA1 levels are genetically co-determined, this IL-6-mediated process can further elevate serum Gd-IgA1 levels in patients with IgAN [11–13].

Genome-Wide Association Studies (GWAS) of multi-ethnic cohorts uncovered multiple candidate genes involved in mucosal immunity that are associated with the development of IgAN [14–16]. Some of these genes, such as *ITGAM* and *TNFSF13*, encode proteins regulating mucosal lymphoid tissues involved in IgA production. A subset of patients with IgAN have elevated serum levels of A Proliferation-Inducing Ligand (APRIL), a cytokine from Tumor-Necrosis Factor (TNF) ligand superfamily member 13 encoded by the *TNFSF13* gene. Furthermore, Toll-like Receptor 9 (TLR9) may be involved in the pathogenesis of IgAN *via* APRIL pathway that affect maturation of plasma cells [17]. Recent clinical trials have reported that administration of an inhibitor of APRIL, TACI-IgG Fc fusion protein (Atacicept), to IgAN patients decreased serum levels of Gd-IgA1 and improved proteinuria [18]. Similarly, a humanized IgG2 monoclonal antibody (Sibeprenlimab), a neutralizing antibody for APRIL, has been also reported to decrease serum levels of Gd-IgA1 and improve proteinuria [19].

Another IgAN-associated locus is the *HORMAD2* locus that contains several genes, including *LIF* and *OSM* that encode cytokines called Leukemia Inhibitory Factor (LIF) and Oncostatin M (OSM), respectively. Furthermore, this GWAS locus is associated with IgAN as well as serum IgA levels and tonsillectomy [20–22]. A recent study postulated that this locus is likely involved in the development of IgAN in association with TLR9 pathways [23]. LIF is an IL-6-related cytokine that uses gp130 for signal transduction and has been previously implicated in mucosal immunity [24] and was identified as a potential drug target [20]. Prior studies of LIF/OSM cytokines revealed that LIF stimulation of immortalized IgA1-producing cell lines derived from peripheral blood of IgAN patients increased Gd-IgA1 production [25]. Follow-up analyses of the signaling mechanisms implicated STAT1-mediated abnormal activation of Src-family kinases, including *Lyn* [26]. *Lyn*, identified in LIF-mediated signaling abnormalities in peripheral blood-derived IgA1-secreting cell lines from IgAN patients, is a non-receptor kinase with a key signaling role in inflammation. The corresponding gene, *LYN*, was recently identified as one of the 16 new IgAN-associated GWAS loci [20].

In summary, we identified that the signaling *via* the LIF/JAK2/STAT1 pathway is involved in LIF-mediated Gd-IgA1 overproduction by immortalized IgA1-producing cell lines derived from tonsils of patients with IgAN [27]. Notably, studies of peripheral-blood mononuclear cells and kidney tissues indicated that enhanced activation of STAT1 in IgAN patients may affect the kidney function [28].

JAK/STAT is a major pathway that responds to and transduces inflammatory signals from extracellular ligands, such as cytokines and chemokines [29]. GWAS revealed a strong association of the genomic locus that contains *LIF* with the risk of IgAN [15,30]. Furthermore, another GWAS publication revealed that the same locus was associated with acute tonsilitis and chronic inflammation of tonsils leading to tonsillectomy as well as with IgA serum levels [21,22].

## CONCLUSION

The abnormal LIF/JAK2/STAT1 signaling and the elevated production of Gd-IgA1 in tonsillar cells in IgAN patients may play a significant role in disease development and progression. Understanding the mechanisms involved in production of Gd-IgA1 in IgAN will be useful in development of future disease-specific therapies.

## References

[1] Wyatt RJ, Julian BA. IgA nephropathy. N Engl J Med 2013;368(25):2402–24014. 23782179 10.1056/NEJMra1206793

[2] Kiryluk K, Novak J. The genetics and immunobiology of IgA nephropathy. J Clin Invest 2014;124(6):2325–2332. 24892706 10.1172/JCI74475 PMC4089454

[3] Suzuki H, Kiryluk K, Novak J, Moldoveanu Z, Herr AB, Renfrow MB, The pathophysiology of IgA nephropathy. J Am Soc Nephrol 2011;22(10):1795–1803. 21949093 10.1681/ASN.2011050464 PMC3892742

[4] Suzuki H, Fan R, Zhang Z, Brown R, Hall S, Julian BA, Aberrantly glycosylated IgA1 in IgA nephropathy patients is recognized by IgG antibodies with restricted heterogeneity. J Clin Invest 2009;119(6):1668–1677. 19478457 10.1172/JCI38468 PMC2689118

[5] Zhao N, Hou P, Lv J, Moldoveanu Z, Li Y, Kiryluk K, The level of galactose-deficient IgA1 in the sera of patients with IgA nephropathy is associated with disease progression. Kidney Int 2012;82:790–796. 22673888 10.1038/ki.2012.197 PMC3443545

[6] Berthoux F, Suzuki H, Thibaudin L, Yanagawa H, Maillard N, Mariat C, Autoantibodies targeting galactose-deficient IgA1 associate with progression of IgA nephropathy. J Am Soc Nephrol 2012;23(9):1579–1587. 22904352 10.1681/ASN.2012010053 PMC3431415

[7] Maixnerova D, Ling C, Hall S, Reily C, Brown R, Neprasova M, Galactose-deficient IgA1 and the corresponding IgG autoantibodies predict IgA nephropathy progression. PloS One 2019;14:e0212254. 30794576 10.1371/journal.pone.0212254 PMC6386256

[8] Horie A, Hiki Y, Odani H, Yasuda Y, Takahashi M, Kato M, IgA1 molecules produced by tonsillar lymphocytes are under-O-glycosylated in IgA nephropathy. Am J Kidney Dis 2003;42:486–496. 12955676 10.1016/s0272-6386(03)00743-1

[9] Inoue T, Sugiyama H, Hiki Y, Takiue K, Morinaga H, Kitagawa M, Differential expression of glycogenes in tonsillar B lymphocytes in association with proteinuria and renal dysfunction in IgA nephropathy. Clin Immunol 2010;136(3):447–455. 20538527 10.1016/j.clim.2010.05.009

[10] Yamada K, Huang ZQ, Raska M, Reily C, Anderson JC, Suzuki H, Inhibition of STAT3 signaling reduces IgA1 autoantigen production in IgA nephropathy. Kidney Int Rep 2017;2(6):1194–1207. 29270528 10.1016/j.ekir.2017.07.002 PMC5733772

[11] Gharavi AG, Moldoveanu Z, Wyatt RJ, Barker CV, Woodford SY, Lifton RP, Aberrant IgA1 glycosylation is inherited in familial and sporadic IgA nephropathy. J Am Soc Nephrol 2008;19(5):1008–1014. 18272841 10.1681/ASN.2007091052 PMC2386728

[12] Kiryluk K, Li Y, Moldoveanu Z, Suzuki H, Reily C, Hou P, GWAS for serum galactose-deficient IgA1 implicates critical genes of the O-glycosylation pathway. PLoS Genet 2017;13(2):e1006609. 28187132 10.1371/journal.pgen.1006609 PMC5328405

[13] Gale DP, Molyneux K, Wimbury D, Higgins P, Levine AP, Caplin B, Galactosylation of IgA1 is associated with common variation in C1GALT1. J Am Soc Nephrol 2017;28(7):2158–2166. 28209808 10.1681/ASN.2016091043 PMC5491291

[14] Feehally J, Farrall M, Boland A, Gale DP, Gut I, Heath S, HLA has strongest association with IgA nephropathy in genome-wide analysis. J Am Soc Nephrol 2010;21:1791–1797. 20595679 10.1681/ASN.2010010076 PMC3013538

[15] Gharavi AG, Kiryluk K, Choi M, Li Y, Hou P, Xie J, Genome-wide association study identifies susceptibility loci for IgA nephropathy. Nat Genet 2011;43:321–327. 21399633 10.1038/ng.787 PMC3412515

[16] Yu XQ, Li M, Zhang H, Low HQ, Wei X, Wang JQ, A genome-wide association study in Han Chinese identifies multiple susceptibility loci for IgA nephropathy. Nat Genet 2012;44(2):178–182. 10.1038/ng.1047 22197929

[17] Muto M, Manfroi B, Suzuki H, Joh K, Nagai M, Wakai S, Toll-like receptor 9 stimulation induces aberrant expression of a proliferation-inducing ligand by tonsillar germinal center B cells in IgA nephropathy. J Am Soc Nephrol 2017;28(4):1227–1238. 27920152 10.1681/ASN.2016050496 PMC5373453

[18] Barratt J, Tumlin J, Suzuki Y, Kao A, Aydemir A, Pudota K, Randomized Phase II JANUS Study of Atacicept in patients with IgA nephropathy and persistent Proteinuria. Kidney Int Rep 2022;7(8):1831–1841. 35967104 10.1016/j.ekir.2022.05.017 PMC9366370

[19] Mathur M, Barratt J, Chacko B, Chan TM, Kooienga L, Oh KH, A Phase 2 trial of sibeprenlimab in patients with IgA nephropathy. Kidney Int Rep 2023. DOI: 10.1056/NEJMoa230563. PMC7615905 37916620

[20] Kiryluk K, Sanchez-Rodriguez E, Zhou XJ, Zanoni F, Liu L, Mladkova N, Genome-wide association analyses define pathogenic signaling pathways and prioritize drug targets for IgA nephropathy. Nat Genet 2023. 1091–1105. 37337107 10.1038/s41588-023-01422-x PMC11824687

[21] Feenstra B, Bager P, Liu X, Hjalgrim H, Nohr EA, Hougaard DM, Genome-wide association study identifies variants in HORMAD2 associated with tonsillectomy. J Med Genet 2017;54(5):358–364. 27941131 10.1136/jmedgenet-2016-104304

[22] Liu L, Khan A, Sanchez-Rodriguez E, Zanoni F, Li Y, Steers N, Genetic regulation of serum IgA levels and susceptibility to common immune, infectious, kidney, and cardio-metabolic traits. Nat Commun 2022;13(1):6859. 36369178 10.1038/s41467-022-34456-6 PMC9651905

[23] Wang YN, Gan T, Qu S, Xu LL, Hu Y, Liu LJ, MTMR3 risk alleles enhance Toll Like Receptor 9-induced IgA immunity in IgA nephropathy. Kidney Int 2023;104(3):562–576. 37414396 10.1016/j.kint.2023.06.018

[24] Cella M, Fuchs A, Vermi W, Facchetti F, Otero K, Lennerz JK, A human natural killer cell subset provides an innate source of IL-22 for mucosal immunity. Nature 2009;457(7230):722–725. 18978771 10.1038/nature07537 PMC3772687

[25] Person T, King RG, Rizk DV, Novak J, Green TJ, Reily C. Cytokines and production of aberrantly O-glycosylated IgA1, the main autoantigen in IgA nephropathy. J Interferon Cytokine Res 2022;42:301–315. 35793525 10.1089/jir.2022.0039 PMC9536348

[26] Yamada K, Huang ZQ, Raska M, Reily C, Anderson JC, Suzuki H, Leukemia inhibitory factor signaling enhances production of galactose-deficient IgA1 in IgA nephropathy. Kidney Dis (Basel) 2020;6:168–180. 32523959 10.1159/000505748 PMC7265702

[27] Yamada K, Huang ZQ, Reily C, Green TJ, Suzuki H, Novak J, LIF/JAK2/STAT1 signaling enhances production of galactose-deficient IgA1 by IgA1-producing cell lines derived from tonsils of patients with IgA nephropathy. Kidney Int Rep 2023. DOI: 10.1016/j.ekir.2023.11.003. PMC10851019 38344714

[28] Tao J, Mariani L, Eddy S, Maecker H, Kambham N, Mehta K, JAK-STAT activity in peripheral blood cells and kidney tissue in IgA nephropathy. Clin J Am Soc Nephrol 2020;15:973–982. 32354727 10.2215/CJN.11010919 PMC7341773

[29] O’Shea JJ, Plenge R. JAK and STAT signaling molecules in immunoregulation and immune-mediated disease. Immunity 2012;36(4):542–550. 22520847 10.1016/j.immuni.2012.03.014 PMC3499974

[30] Kiryluk K, Li Y, Scolari F, Sanna-Cherchi S, Choi M, Verbitsky M, Discovery of new risk loci for IgA nephropathy implicates genes involved in immunity against intestinal pathogens. Nat Genet 2014;46(11):1187–1196. 25305756 10.1038/ng.3118 PMC4213311

